# Recent Advances in Developing Small Molecules Targeting Nucleic Acid

**DOI:** 10.3390/ijms17060779

**Published:** 2016-05-30

**Authors:** Maolin Wang, Yuanyuan Yu, Chao Liang, Aiping Lu, Ge Zhang

**Affiliations:** 1 Institute of Integrated Bioinfomedicine and Translational Science (IBTS), School of Chinese Medicine, Hong Kong Baptist University, Hong Kong 999077, China; wangml1240@163.com (M.W.); yu.yy01@hotmail.com (Y.Y.); liangchao512@163.com (C.L.); 2 Shenzhen Lab of Combinatorial Compounds and Targeted Drug Delivery, HKBU Institute of Research and Continuing Education, Shenzhen 518000, China; 3 Institute for Advancing Translational Medicine in Bone and Joint Diseases, School of Chinese Medicine, Hong Kong Baptist University, Hong Kong 999077, China

**Keywords:** nucleic acids, nucleic acids targeting, small molecules, DNA drug discovery, RNA drug discovery

## Abstract

Nucleic acids participate in a large number of biological processes. However, current approaches for small molecules targeting protein are incompatible with nucleic acids. On the other hand, the lack of crystallization of nucleic acid is the limiting factor for nucleic acid drug design. Because of the improvements in crystallization in recent years, a great many structures of nucleic acids have been reported, providing basic information for nucleic acid drug discovery. This review focuses on the discovery and development of small molecules targeting nucleic acids.

## 1. Introduction

Nucleic acids play significant roles in variety kinds of biological processes [1,2,3,4,5]. According to the differences on sugar scaffold, nucleic acids can be classified into two categories. DNAs and RNAs participate in gene storage, replication, transcription and other important biological activities. Thus, targeting nucleic acids can regulate a large range of biological processes, especially genetic diseases. Nucleic acids play significant roles in anticancer [6] and antiviral [7] processes. Therefore, the small molecules targeting nucleic acids are desirable and have become a topic issue in recent years.

However, small molecules targeting nucleic acids are more difficult to discover than targeting protein, which result from various reasons. Nucleic acid lacks spatial structure information from X-ray crystallization, nuclear magnetic resonance (NMR) or other imaging methods. Current approaches for small molecules targeting protein are incompatible with nucleic acids. Most of the docking methods, for example, do not regard RNA and DNA receptors as flexible bodies [8,9,10], which constrains the conformational changes of nucleic acids. Fortunately, the areas of structural biology and computational chemistry have improved dramatically. A large number of structures of nucleic acids have been reported. Several computational methods and force fields have been developed and modified [11,12]. These improvements provide a new horizon to discovery and design novel small molecules targeting nucleic acids.

Depending on the differences on sugar scaffold of nucleic acids, small molecules targeting nucleic acids can be separated into two main categories: small molecules targeting DNA and small molecules targeting RNA (in which DNA and RNA can form different conformations). This review focuses on the recent discovery and development of small molecules targeting DNA and RNA.

## 2. Small Molecules Targeting DNA

### 2.1. Small Molecules Targeting DNA Duplex

#### 2.1.1. Small Molecules Forming Covalent Bonds with DNA Duplex

**Figure 1:**
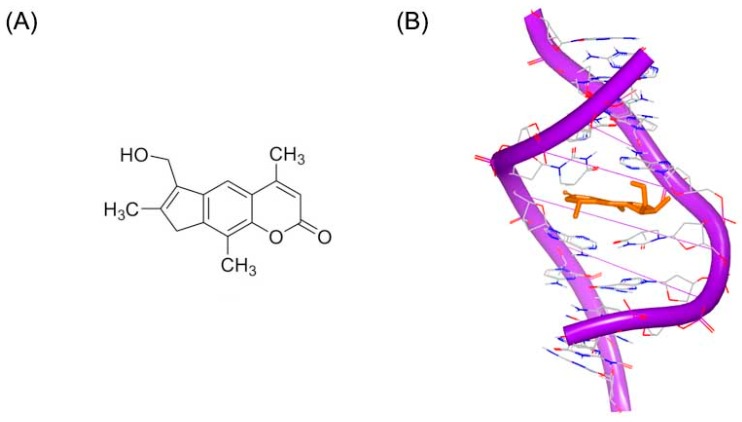
(**A**) Chemical structure of HMT; and (**B**) NMR structure of a short DNA complex with HMT (RCSB Protein Data Bank (PDB) code: 204D). The orange structure represents the small molecule. The purple structure represents the backbone of nucleic acid; the blue and red atoms in the molecule represent nitrogen and oxygen atoms, respectively.

**Figure 2:**
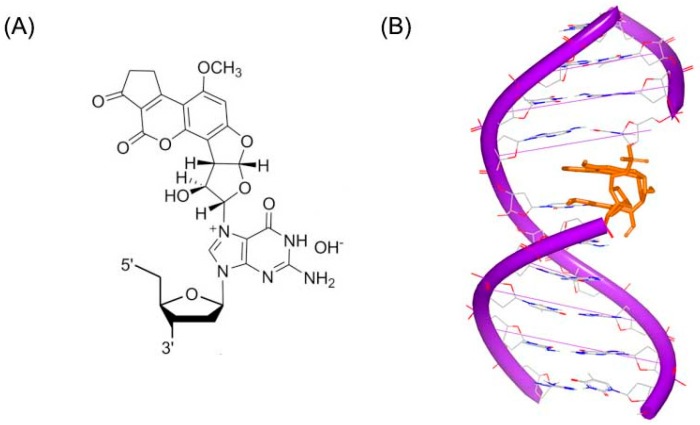
(**A**) Chemical structure of Aflatoxin B1 adduct; and (**B**) NMR structure of a DNA duplex complex with Aflatoxin B1 adduct (PDB code: 2KH3). The orange structure represents the small molecule; the purple structure represents the backbone of nucleic acid; the blue and red atoms in the molecule represent nitrogen and oxygen atoms, respectively.

Psoralen or psoralene has been used as mutagen to treat skin diseases including psoriasis and vitiligo. Psoralen interacts with standard DNA duplex, through the 5,6 double bond in pyrimidine ring, which can stop replication and transcription. 4′-(Hydroxymethyl)-4,5′,8-trimethylpsoralen, short for HMT, has reasonable water solubility and high DNA binding affinity, as shown in Figure 1A. Figure 2B shows the crystal structure of a short DNA complex with HMT reported by Spielmann *et al.* [13]. The HMT structure crosses both the minor and major grooves through the 5,6 double bonds of pyrimidine ring and two thymines. There are apparent stacking interactions from the ring system of HMT and the bases nearby. The high binding affinity of HMT and duplex results from aromatic rings stacking and hydrophobic interactions. The crystal study of the complex of DNA and HMT showed the Holliday junction was formed in the adduct of DNA-HMT. The interaction between DNA and HMT is related with sequence, which may play a role in repairing the psoralen damage in chemotherapy treatment. The conformation of DNA is extremely twisted at the bases of thymine connected with the hexatomic pyrone of the molecule.

Aflatoxin B1 (AFB) is a common contaminant in a variety of foods including peanuts, cottonseed meal, corn, and other grains as well as animal feeds. Aflatoxin B1 is considered the most toxic aflatoxin and it is highly implicated in hepatocellular carcinoma (HCC) in humans [14]. The epoxide metabolite of AFB can react with duplex DNA to produce a cationic adduct (Figure 2A). An NMR structure of this adduct has been resolved and reported by Stone [15], as shown in Figure 2B. This NMR structure shows that Aflatoxin B1 substructure inserts into DNA duplex strand. The adduct acts as a covalent binder, which can crosslink to duplex DNA conformation inducing the modification and change in the DNA conformation. The changed DNA will stop interacting with related proteins, which can block the process of replication or transcription.

**Figure 3:**
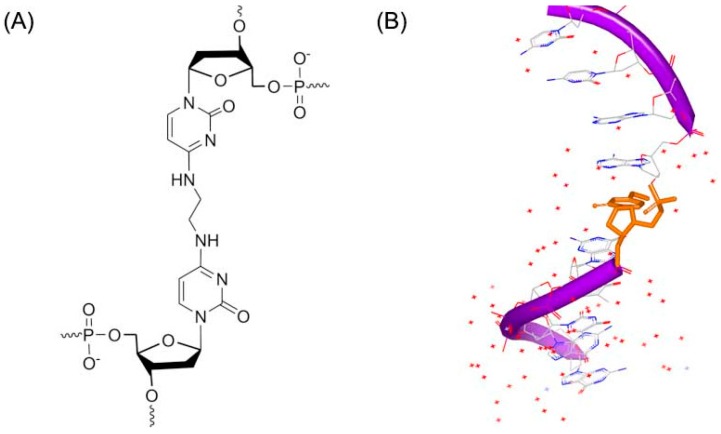
(**A**) Chemical structure of N^4^C–ethyl–N^4^C adduct; and (**B**) one strand of NMR structure of a DNA duplex complex with N^4^C–ethyl–N^4^C adduct (PDB code: 2OKS). The orange structure represents the small molecule; the purple structure represents the backbone of nucleic acid; the blue and red atoms in the molecule represent nitrogen and oxygen atoms, respectively.

Another DNA duplex adduct, a synthetic N^4^C–ethyl–N^4^C (Figure 3A), was discovered to interact with DNA by Miller [16]. The DNA study indicated that the ethyl cross-linked the base pairs in the structure solution. However, the ethyl linker does not remarkably change the B-form DNA conformation [17], as shown in Figure 3B.

**Figure 4:**
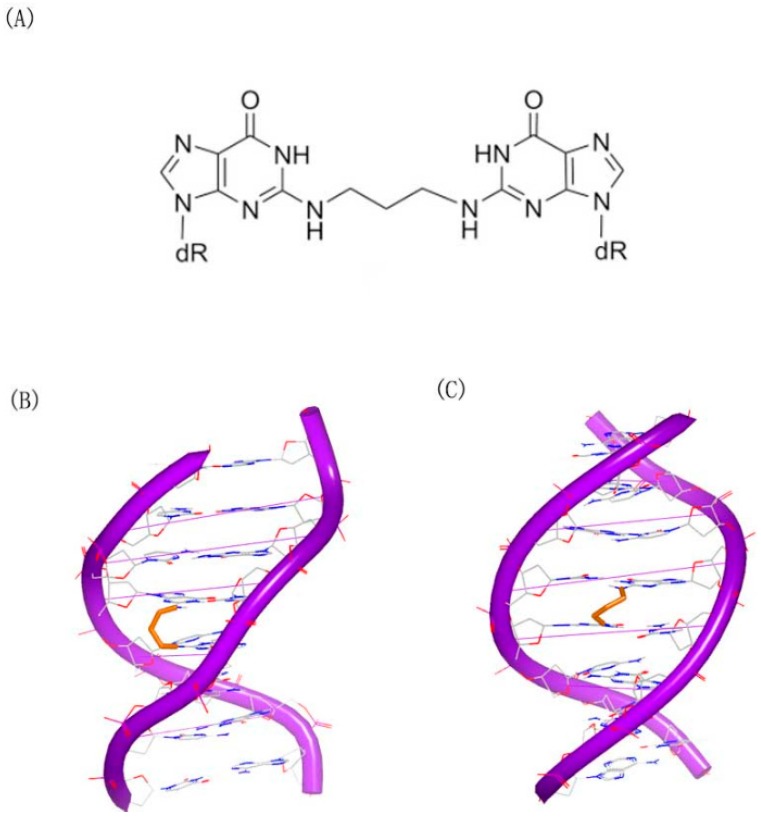
(**A**) Chemical structure of N^2^G–trimethylene–N^2^G; (**B**) crystal complex of a DNA with N^2^G–trimethylene–N^2^G cross-link (PDB code: 2KNK); and (**C**) crystal complex of a DNA with N^2^G–trimethylene–N^2^G cross-link (PDB code: 2KNL). The orange structure represents the small molecule; the purple structure represents the backbone of nucleic acid; the blue and red atoms in the molecule represent nitrogen and oxygen atoms, respectively.

Similar interactions were reported in the trimethylene related structures (Figure 4A) and DNA duplex conformation [18]. Two DNA duplexes structures were resolved and submitted to Protein Data Bank. Although the stacking between the linked bases and neighbor bases was different, both of the two structures indicated that the duplexes could maintain the hydrogen bonds and the B-form geometry (Figure 4B,C).

**Figure 5:**
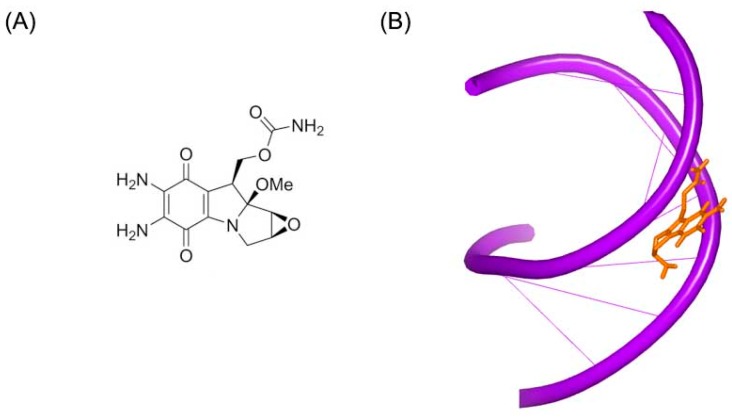
(**A**) Chemical structure of mitomycin C; and (**B**) crystal complex of a short DNA with mitomycin C (PDB code: 199D). The orange structure represents the small molecule; the purple structure represents the backbone of nucleic acid.

Mitomycin C (Figure 5A) is a member of mitomycins, which contains aziridine substructure and are extracted from natural products isolated from streptomycetes. The molecule is used as a chemotherapy drug for cancer. Mitomycin C alkylates the guanine in C–G base pairs of the DNA conformation [19]. The crystal complex of a short DNA with mitomycin C was reported by Patel *et al.* [20]. Unlike other standard DNA duplexes, the mitomycin alkylated DNA duplex shows A-form base pair stacking and B-form sugar puckers. The ring of mitomycin locates in the minor groove. And the ring of indoloquinone forms a 45° to the helix axis, as shown in Figure 5B.

As mentioned, these small molecules can modify and change the overall conformation of the conformation by cross-linking to DNA duplexes. The changed DNA conformations will stop interacting with the corresponding biological partners, thus the transcription or replication process will be blocked. This is how this kind of molecules works in the chemotherapeutics treatment. These molecules showed high toxicity in normal cells, just because of its low selectivity. To decrease resistance and promote selectivity, the comprehension of the interaction mechanism between DNA and drugs will be of significant importance.

#### 2.1.2. Small Molecules Targeting with DNA Duplex in Minor Groove

**Figure 6:**
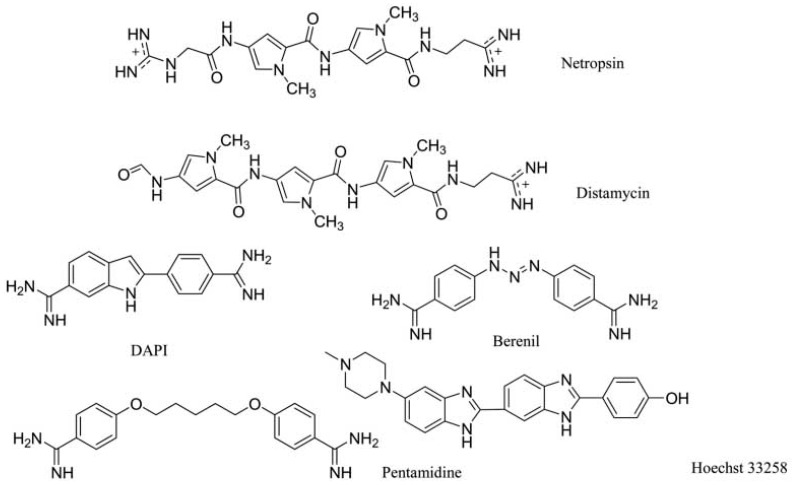
Typical structures of small molecules for DNA minor groove.

**Figure 7:**
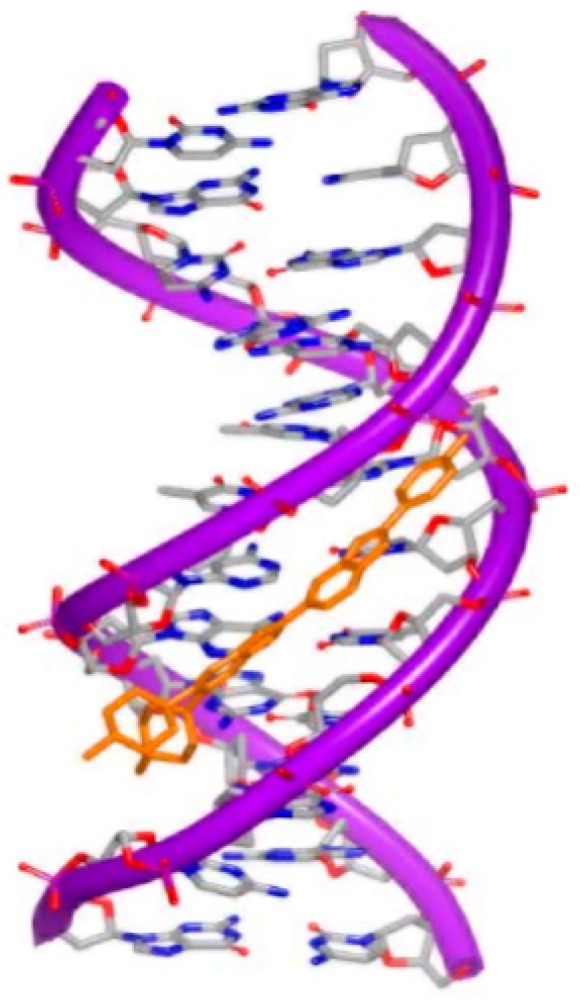
Crystal complex of DNA duplex and Hoechst 33528 (PDB code: 8BNA). The orange structure represents the small molecule; the purple structure represents the backbone of nucleic acid; the blue and red atoms in the molecule represent nitrogen and oxygen atoms, respectively.

To affect the gene expression, the small molecules can not only insert into the DNA strand, but also bind to the major or minor grooves of high binding affinity. For minor groove, typical small molecules are heterocylic dications and polyamides, including netropsin, berenil, and pentamidine, as shown in Figure 6. Originally, these molecules were found to bind AT-rich areas preferentially. Then, these molecules appeared near G–C and C–G base pairs [21,22]. The molecules targeting minor groove have been studied as a series of therapeutics with anti-viral, anti-tumor and anti-bacterial activities [23,24,25]. Some structural studies have been investigated as the basis of the design of small molecules [26,27,28,29,30]. The crystal complex of DNA duplex and Hoechst 33528 is shown in Figure 7. The Hoechst 33528 is clamped in the minor groove of the DNA structure.

**Figure 8:**
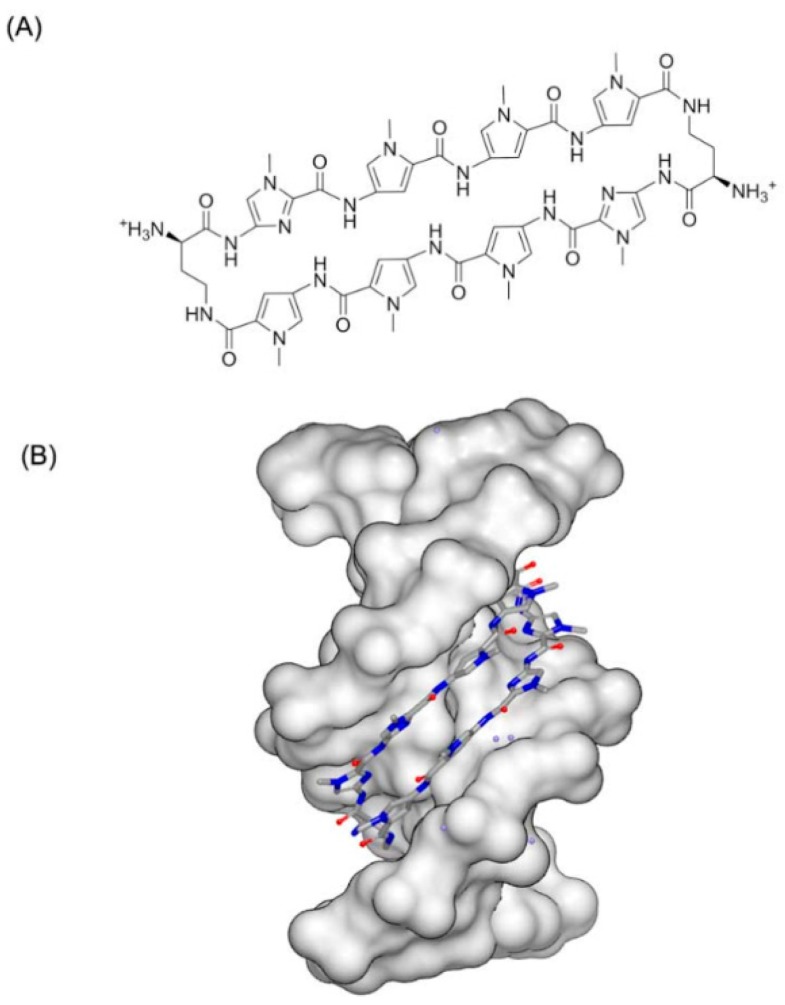
(**A**) Chemical structure of 8 ringmolecule; and (**B**) complex of a DNA with 8 ring molecule (PDB code: 3I5L). The blue and red atoms in the molecule represent nitrogen and oxygen atoms, respectively.

Except for the drug-like molecules, some unusual compounds were also identified to locate in the minor groove of DNA. An 8 ring molecule (Figure 8A) was reported that it can bind to a DNA duplex [31]. The resolved structure indicates that the DNA conformation has been remarkably changed. The minor groove has been widened, and the major groove has been narrowed. The minor and major grooves are both approximate 4 Å (Figure 8B). Additionally, the helix center direction is twisted more than 18° to the major groove, in comparison with the inherent counterparts. The distortion provides a fundamental element for supporting the concept of allosteric change of the DNA conformation with inhibitors locating in minor grooves.

**Figure 9:**
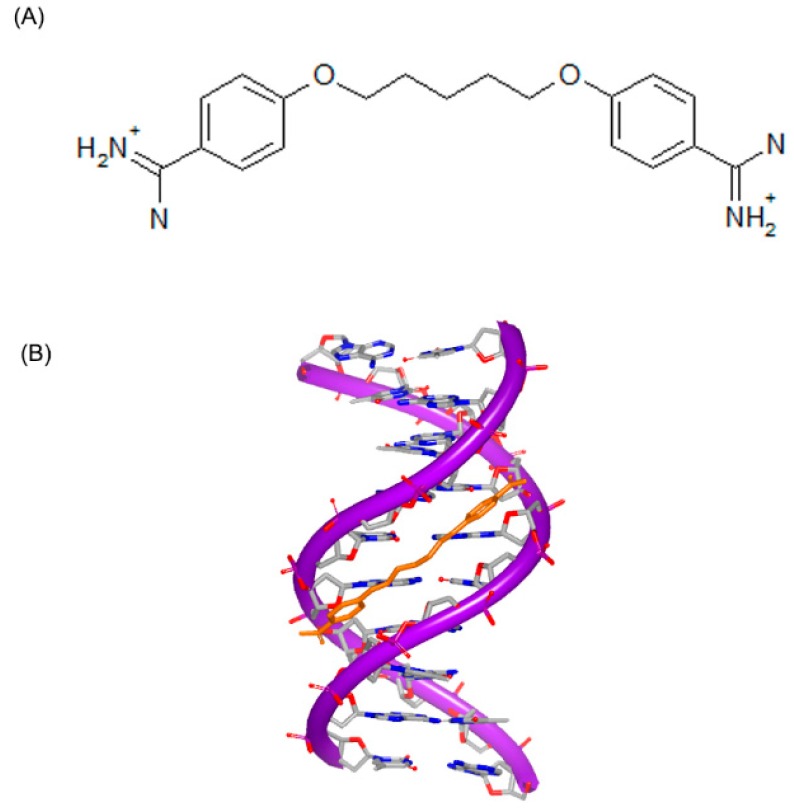
(**A**) Chemical structure of pentamidine; and (**B**) complex of DNA with pentamidine (PDB code: 3EY0). The orange structure represents the small molecule; the purple structure represents the backbone of nucleic acid; the blue and red atoms in the molecule represent nitrogen and oxygen atoms, respectively.

The small molecules can bind to nonstandard DNA duplex as well. Hoogsteen base pairs may occur in alternating A–T sequences, which are abundant in eukaryotic animals. Because of the lack of structural information, however, there is little analysis in this field. Recently, the complex of Hoogsteen DNA duplex and pentamidine (Figure 9A) was determined and reported [32]. The X-ray DNA structure presents a mixture of Watson–Crick and Hoogsteen pattern (Figure 9B). Unlike previous minor binders, pentamidine does not bind to the minor groove totally. Only the center of the pentamidine is bound to the minor groove, leaving the positively charged ends detached from the DNA and free to interact with phosphate groups from adjacent duplexes in the crystal. This new binding pattern has potent inspiration for antibacterial design of pentamidine.

#### 2.1.3. Small Molecules Targeting with DNA Duplex in Major Groove

**Figure 10:**
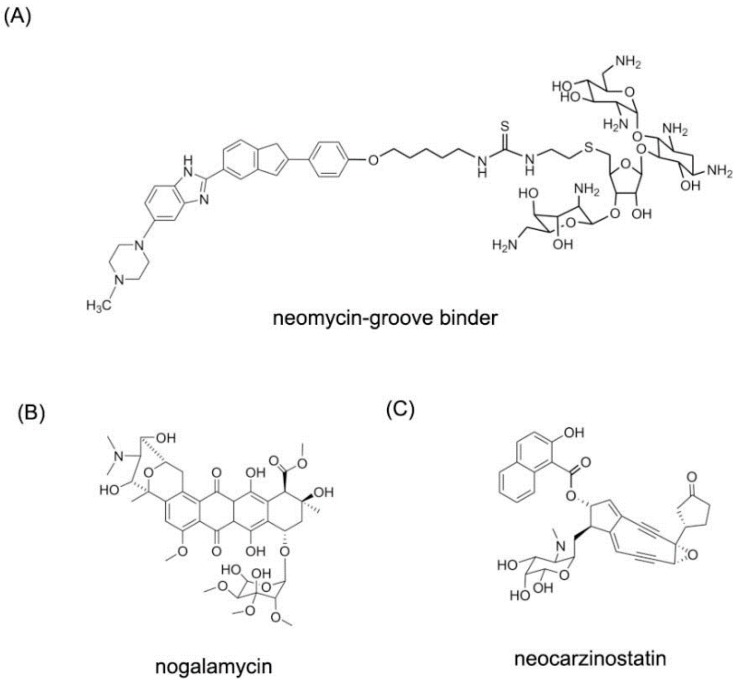
Typical structures of small molecules for DNA major groove. (**A**) The structure of neomycin-grove binder; (**B**) The structure of nogalamycin; (**C**) The structure of neocarzinostatin.

For major groove studies, there are less reports of small molecule targeting information. Due to the requirement of larger compounds, few complexes of molecules and DNA major grooves are determined. Although majority of carbohydrates bind to the DNA minor grooves, some of them can show binding affinity to DNA major groove. The reason for this major groove binding is the large size of carbohydrates and their hydrophilic and hydrophobic substructures. Some classic major groove binders are listed in Figure 10 [33].

**Figure 11:**
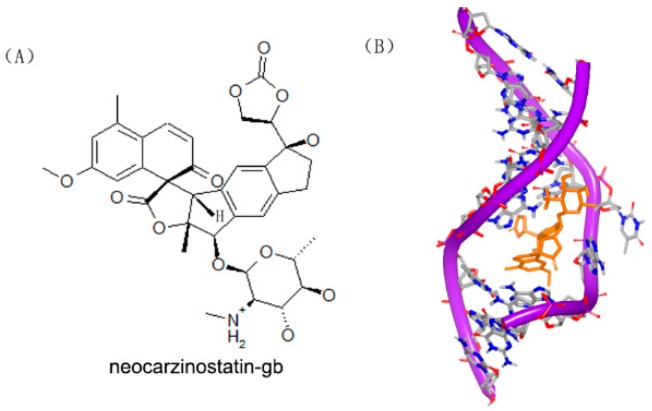

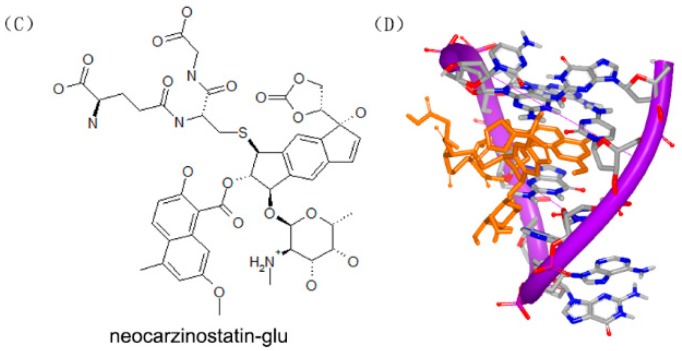
(**A**) Chemical structure of neocarzinostatin-gb; (**B**) complex of a DNA with neocarzinostatin-gb (PDB code: 1KVH); (**C**) chemical structure of neocarzinostatin-glu; and (**D**) complex of DNA with neocarzinostatin-glu (PDB code: 1MP7). The orange structure represents the small molecule; the purple structure represents the backbone of nucleic acid; the blue and red atoms in the molecule represent nitrogen and oxygen atoms, respectively.

Neocarzinostatin and related derivatives have been used as anti-tumor agents for decades [34,35]. This kind of molecule can form a complex with protein and damage DNA by hydrogen abstraction [36]. In order to understand the mechanism and interaction between DNA duplex and molecule, the complex of the DNA duplex and neocarzinostatin were reported. Neocarzinostatin-gb and neocarzinostatin-glu were used as ligand to bind to the DNA duplex. As shown in Figure 11, both of the two complex structures are observed [37,38]. The majority of the neocarzinostatin-gb binds to the major groove. The two rings of the molecule are close to each other in the structure. However, the neocarzinostatin-glu binds to the minor groove. The differences of the two binding modes provide significant inspiration into small molecule-DNA specific targeting.

#### 2.1.4. Small Molecules Intercalating into DNA Duplex

Inserting a molecule between the base pairs of DNA is another binding pattern of small molecules targeting DNA duplexes. Inserting to the base pairs can interrupt DNA replication and transcription. The insertion results from hydrophobic, hydrogen bonding and van der Waals forces [39]. The insertion lengthens the length of DNA and reduces the helical twist [40,41]. In previous study, only molecules with fused ring systems were found to insert into the base pairs of DNA duplexes. Later, molecules with electrophilic or cationic groups behaved the same insertion pattern. However, the ring systems were not required [42,43]. Various compounds have been identified as intercalators, including ditercalinium, dactinomycin, daunomycin and adriamycine. Intercalators can be used as anti-fungal, anti-tumor, and anti-neoplastic agents. However, due to the toxicity, most of the compounds were failed during the clinical trial [44].

**Figure 12:**
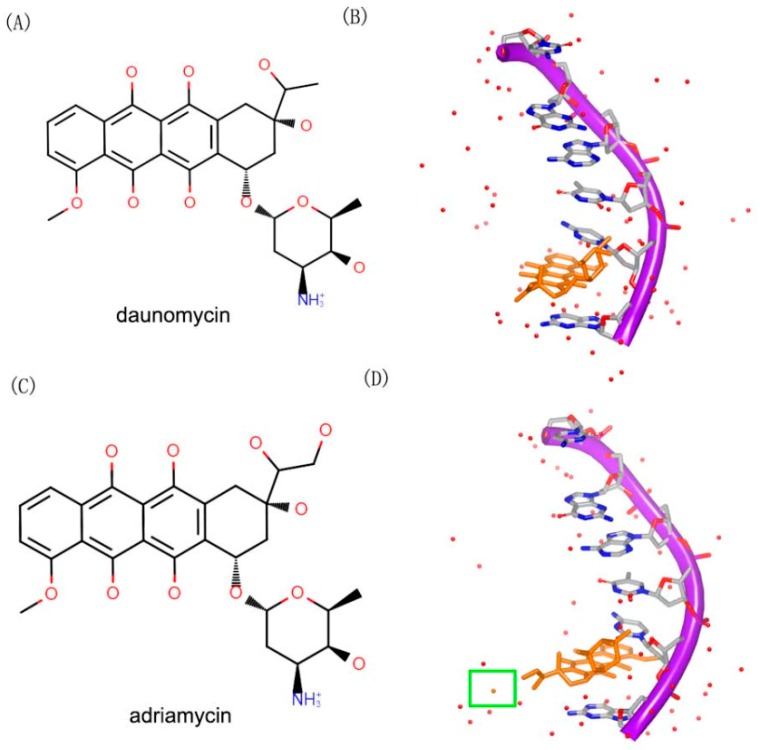
(**A**) Chemical structure of daunomycin; (**B**) one strand of a DNA duplex complex with daunomycin (PDB code: 1D10); (**C**) chemical structure of adriamycin; and (**D**) one strand of a DNA duplex complex with adriamycin (PDB code: 1D12). The orange structure represents the small molecule; the purple structure represents the backbone of nucleic acid; the red dots represent the water molecules; the blue and red atoms in the molecule represent nitrogen and oxygen atoms, respectively. The green square represents the water molecule that interacts with the small molecule.

Daunomycin (trade name Cerubidine) is used to treat various cancers as chemotherapy drug [45]. Specifically, the most common use is treating some types of leukemia. Daunomycin (Figure 12A) consists of a planar ring, an amino sugar structure and a fused cyclohexane ring system. A lot of structural studies have been investigated to understand the interaction between DNA duplex and molecule [28,46,47,48,49,50,51]. Most of the structures indicate that two daunomycin molecules insert into the G–C steps with the sugar moiety in duplex, as shown in Figure 12B. Through sequence selectivity investigation by different biophysical methods, it was revealed that this ligand prefers a purine-pyrimidine step and the DNA duplexes are usually elongated or twisted after binding to the small molecule.

Adriamycin (Figure 12C) contains an extra hydroxyl group, compared to daunomycin. Although, the daunomycin and adriamyc in have nearly the same structure, their functions are remarkably diverse. Adriamycin is used to treat the tumor, while daunomycin is an effective drug in leukemias [47]. The complex structures of DNA duplex and compounds have been determined. In order to understand the differences between the two compounds, the complexes of the two molecules with a same DNA were resolved. The two complex structures showed a little differences between each other. Moreover, the hydroxyl group of the adriamycin interacts with a water molecule in the complex (Figure 12D).

**Figure 13:**
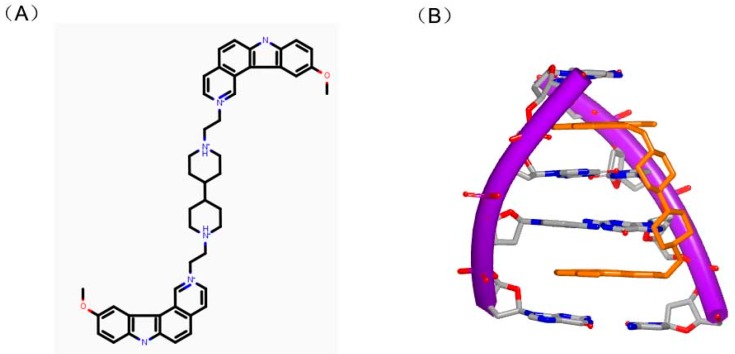
(**A**) Chemical structure of dimer of ditercalinium; and (**B**) complex of a DNA duplex with ditercalinium (PDB code: 1D32). The orange structure represents the small molecule; the purple structure represents the backbone of nucleic acid; the blue and red atoms in the molecule represent nitrogen and oxygen atoms, respectively.

Ditercalinium, as shown in Figure 13A, is regarded as a DNA intercalator in treatment of cancer. The structure of ditercalinium is a dimer of pyridocarbozole. Thus, the molecule can bind to DNA duplex by bis-intercalation and then induce DNA repair in eukaryotic or prokaryotic cell [52,53,54]. Afterwards, pyridocarbozole derivatives and related bioactivity have been studied [55,56,57]. The structure of DNA duplex and ditercalinium showed that the dimer of the molecule inserted to two G–C steps of the conformation (Figure 13B). The duplex of the DNA maintains a right-handed helix and has a binding site in major groove. The nitrogens with positive charge of the molecule make the charge interaction toward major groove of DNA [58]. In the complex structure, the helix is twisted at approximate 15° to minor groove of DNA. The mentioned structural features give inspiration on further research of optimizing this kind of molecules.

**Figure 14:**
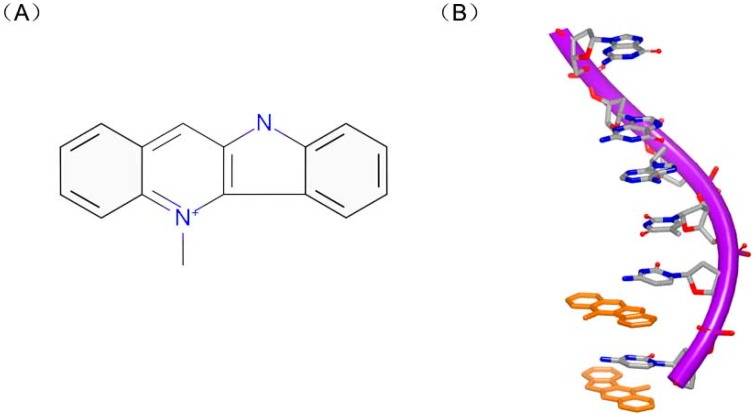
(**A**) Chemical structure of dimer of cryptolepine; and (**B**) one strand of a DNA duplex complex with cryptolepine (PDB code: 1K9G). The orange structure represents the small molecule; the purple structure represents the backbone of nucleic acid; the blue and red atoms in the molecule represent nitrogen and oxygen atoms, respectively.

Actually, the specificity of DNA intercalators is the reason for drug toxicity. Molecules can bind to unexpected DNA sequence for new treatment. Cryptolepine (Figure 14A) is a good example for occasional drug discovery. Cryptolepine is isolated from *Cryptolepis triangular* and used as anti-malarial and cytotoxic agent [59]. This compound can insert with C–G rich sequences [60]. The crystal complex structure indicates that the drug interacts with the duplex in a base stacking insertion pattern (Figure 14B). The molecule and two consecutive C–G base pairs form a sandwich conformation with two C–G rich sequences. And the hexatomic ring stacks in the middle of cytosine and guanine. Structurally, cryptolepine was mildly twisted at about 6.8° between two aromatic rings. Besides, the charged nitrogen can improve the stability of the complex with the interaction between molecule and DNA duplex. The insertion into base pair steps and the molecular dissymmetry were important elements for this interaction.

#### 2.1.5. Small Molecules Targeting with DNA Duplex through Multiple Binding Patterns

**Figure 15:**
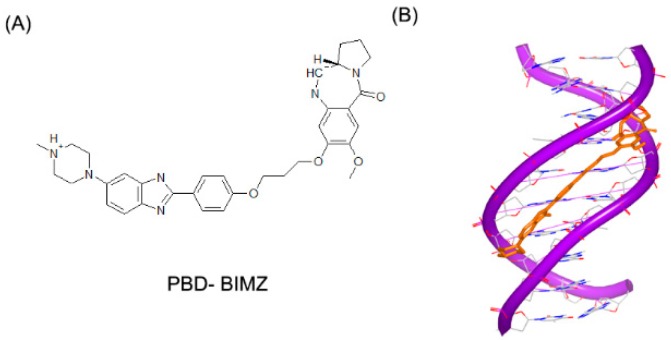

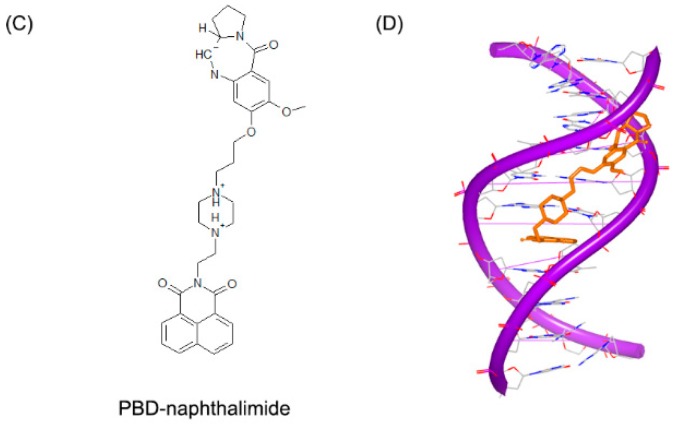
(**A**) Chemical structure of PBD-BIMZ; (**B**) complex of a DNA duplex with PBD-BIMZ (PDB code: 2KTT); (**C**) chemical structure of PBD-naphthalimide; and (**D**) complex of a DNA duplex with PBD-naphthalimide (PDB code: 2KY7). The orange structure represents the small molecule; the purple structure represents the backbone of nucleic acid; the blue and red atoms in the molecule represent nitrogen and oxygen atoms, respectively.

Small molecules can not only bind to DNA duplex in a single mode, but also interact with DNA duplex in multiple binding patterns, which will make the complex more stable [61,62]. PBD-BIMZ (Figure 15A) binds to a DNA duplex in a covalent link to a guanine base. The complex shows that the hybrid molecule orients in the minor groove (Figure 15B) [63]. Although the covalent binding distorts the DNA helix, the overall duplex still maintains the standard B-form conformation. It is revealed that covalent binding site in local region possesses specific twisted helix. By comparison, the piperazine ring shows a high flexibility with various conformations. Later, the same group reported another ligand (Figure 15C) in complex with the same DNA duplex. This hybrid compound binds to a guanine in a covalent link and the naphthalimide substructure embeds to (A–T)_2_ steps, as shown in Figure 15D [64]. The covalent binding will remarkably make the DNA and ligand stable. Moreover, because many molecules have low specificity for sequence, the multiple patterns can make the molecules sequence targetable.

### 2.2. Small Molecules Targeting DNA Triplex

Single strand DNA can bind to a DNA duplex structure to form a triplex conformation DNA [65]. The interactions between strands are Hoogsteen or reverse Hoogsteen base pairs. Small molecules can bind to triplex DNA groove, blocking the access of other DNA natural substrate [66]. The DNA triplex can display two forms, intermolecular triplexes and intramolecular triplexes [67]. Intermolecular triplex is generated from a DNA chain of an extra DNA structure. Generally, the intermolecular triplex has attracted attention due to the possible treatment for various cancers or other diseases. In fact, most of the DNA duplex intercalators can insert to the triplex as well [68].

**Figure 16:**
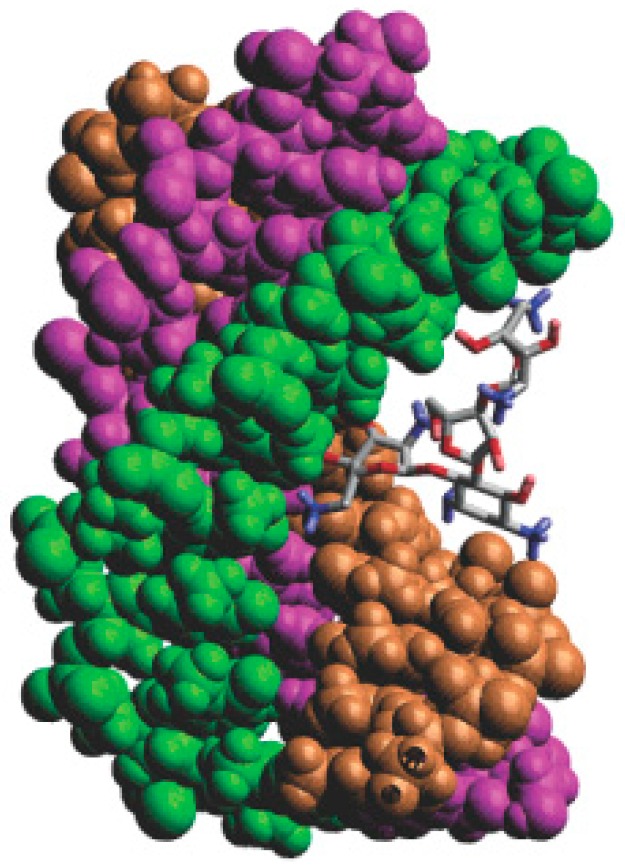
Computer generated model of neomycin bound to a DNA triplex. Ring I of neomycin locates in the middle of the DNA groove, and Ring II and IV facilitate bridging the pyrimidine strands; the orange, green and purple structures represent the three chains of the DNA; the blue and red atoms in the molecule represent nitrogen and oxygen atoms, respectively.

As mentioned above, neomycin is a typical major DNA groove binder. In triplex system, neomycin can make the base mixed DNA triplexes and T–A–T triplex stable. The computational docking conformation [69] of the triplex and neomycin is shown in Figure 16. The computational and biophysical researches both indicate that this Watson–Hoogsteen complementarity leads to the selectivity.

Similar to major groove binders, neomycin conjugates are also found to bind with the DNA triplex structures. Computational modeling of pyrene–neomycin indicates pyrene will insert into the base pairs when neomycin bind with Watson–Hoogsteen groove [70].

### 2.3. Small Molecules Targeting DNA Quadruplex

Telomere regulation has been regarded as a significant potential strategy for cancer therapy. Guanosine rich nucleic acids are regarded as a new class of non-antisense nucleic acids with active G-quartet conformation [71]. Except for telomerase, G-quadruplex-forming sequences have also been found in the promoter regions of many other genes. Small molecules can stabilize G-quartet structure, causing the telomerase low activity [72,73,74]. Therefore, the G-quadruplex structures have attracted more attention for drug design.

**Figure 17:**
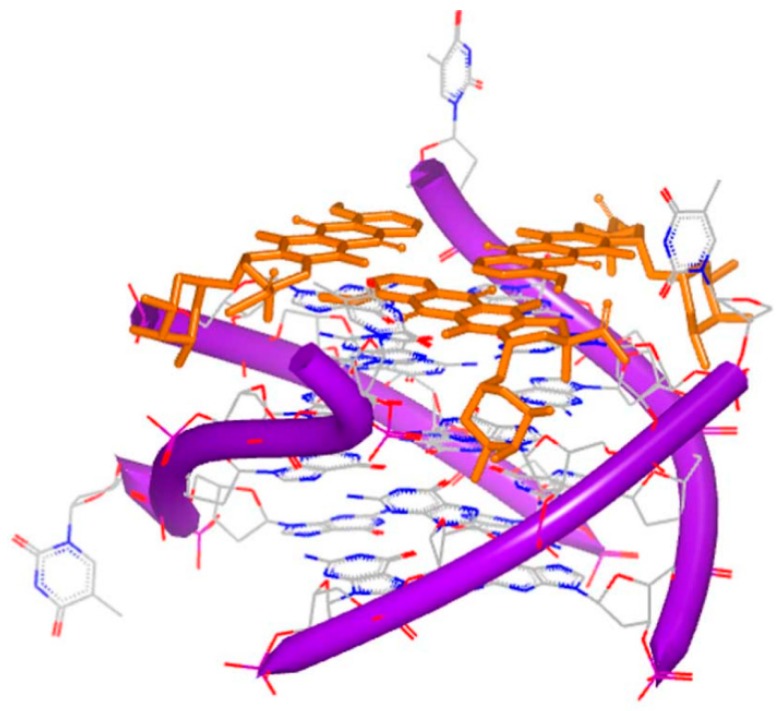
Crystal complex of a DNA quadruplex with daunomycin (PDB code: 1O0K). The orange structure represents the small molecule; the purple structure represents the backbone of nucleic acid; the blue and red atoms in the molecule represent nitrogen and oxygen atoms, respectively.

Because G-quartet conformations consist of planar stacked guanine bases, numbers of small molecules with electron deficient aromatic rings are identified to bind [75]. As mentioned above, distamycin A and daunomycin are typical intercalators for DNA duplex. In quadruplex systems, these compounds are able to bind to DNA quadruplex structures. The first complex of G-quadruplex and daunomycin was determined and reported by Neidle [76]. Figure 17 shows the four parallel strands and three daunomycin molecules at the end of the complex. This complex indicates that the structure shows high stability with the molecules stacking on the end.

**Figure 18:**
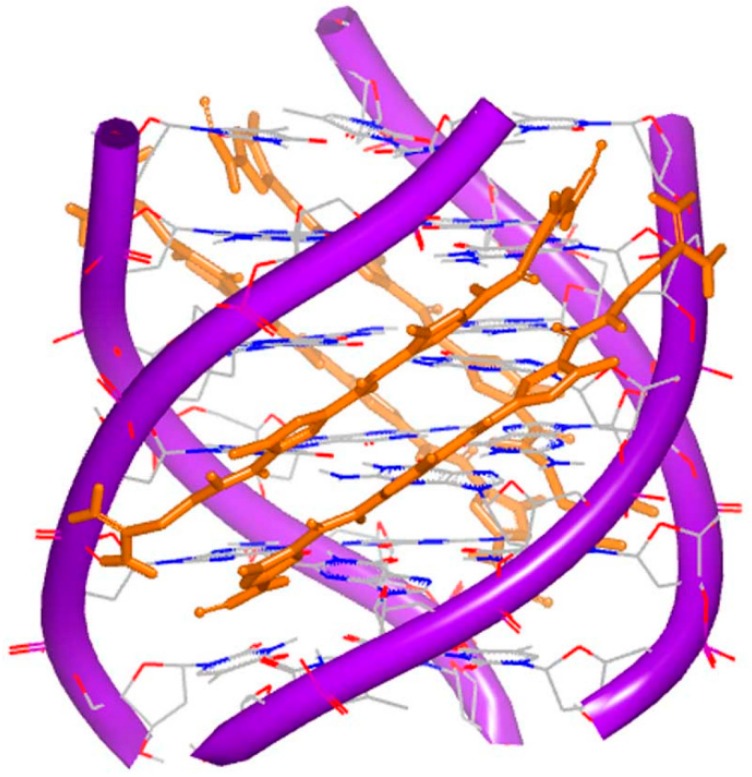
Complex of a DNA quadruplex with distamycin A (PDB code: 2JT7). The orange structure represents the small molecule; the purple structure represents the backbone of nucleic acid; the blue and red atoms in the molecule represent nitrogen and oxygen atoms, respectively.

It is known that distamycin A binds with the minor groove of DNA duplex. The structure of G-quadruplex and distamycin A was determined and reported by Randazzo [77]. The structure indicates that molecule performs the similar binding to DNA duplex minor groove binding. The dimer of the molecule binds at the converse position of the quadruplex conformation (Figure 18). The quadruplex conformation is made up of four identical parallel sequences TGGGGT. At the top of the structure, three stacking daunomycins with sodium ions are located. The quadruplex conformation resembles the inherent counterpart. The structures of daunomycins interact to the DNA grooves by forming hydrogen bonds and van der Waals forces. The complex shows the structure is more stable when the molecules stack at the top than intercalate, because of the large termination of the molecule surface.

**Figure 19:**
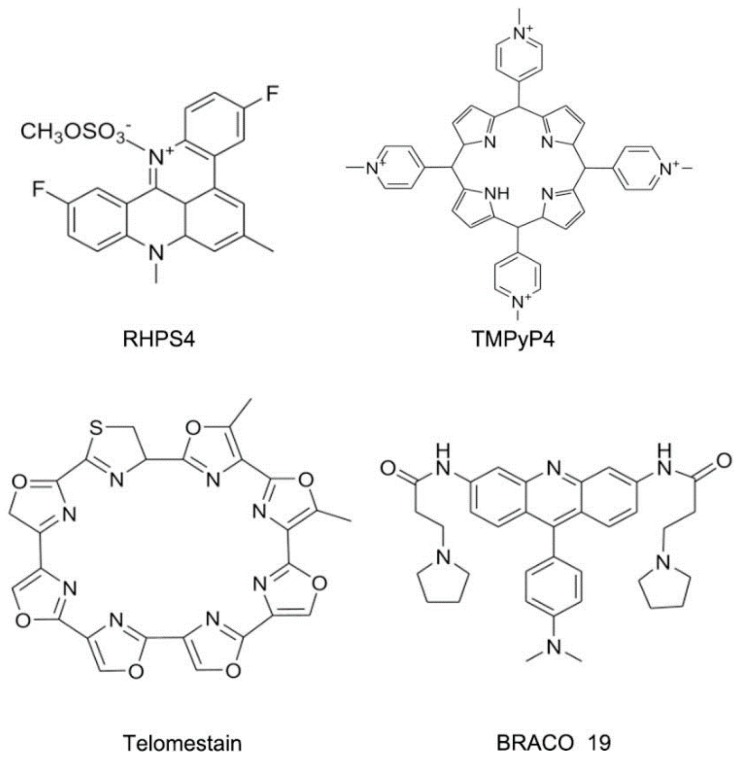
Typical structures of small molecules for DNA quadruplex.

Compared to DNA duplex binding molecules, some novel kinds of molecules have been found. These molecules can bind specifically to DNA quadruplex structures, as shown in Figure 19. The structural and biochemical studies displayed detailed information of the interaction between small molecules and DNA quadruplex. In addition, it also provideda novel platform for further drug design targeting DNA quadruplex. The progress in this field has been reported [78].

## 3. Small Molecules Targeting RNA

### 3.1. Small Molecules Targeting Ribosome

**Figure 20:**
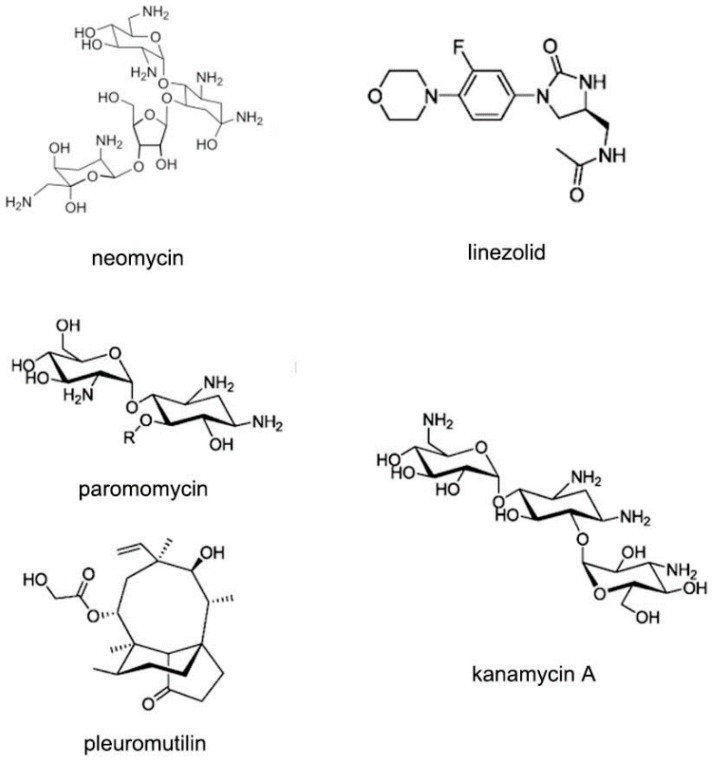
Typical structures of small molecules for 30S or 50S subunit of rRNA.

The ribosomal RNA (rRNA) is the most comprehensively studied among various kinds of RNA types. The ribosome is an attractive anti-bacterial target due to its important function in protein expression [79,80,81,82,83]. The ribosomes in prokaryotic cells mainly contain 30S and 50S subunits. The subunit 30S is for ensuring that the tRNA can locate in the correct position. The subunit 50S is responsible for peptide connection. Various kinds of compounds have been identified to bind the subunits of rRNA, including pleuromutilin, oxazolidinones and aminoglycosides, as shown in Figure 20 [84,85].

**Figure 21:**
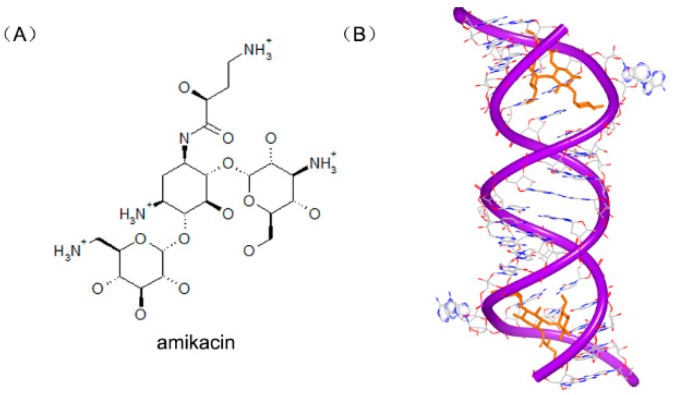
(**A**) Chemical structure of amikacin; and (**B**) complex of a RNA duplex with amikacin (PDB code: 4P20). The orange structure represents the small molecule; the purple structure represents the backbone of nucleic acid; the blue and red atoms in the molecule represent nitrogen and oxygen atoms, respectively.

Amikacin (Figure 21A), a member of aminoglycoside antibiotics, is used to inhibit replication of various kinds of bacteria. Amikacin binds with the 30S subunits of rRNA, leading to the wrong reading of message RNA and making the bacteria cell incapable of translation and growing. The structure of RNA duplex is nearly the same with the previous RNA-aminoglycoside complexes [86,87,88,89,90]. One of the aromatic rings inserts into A site helix by stacking. Another aromatic ring forms four hydrogen bonds to the H-bond acceptors nearby. These common interactions make the amikacin bind to RNA duplex tightly, as shown in Figure 21B [91], and help maintain A1493 and A1492 in the bulge structure, corresponding with the “on” state in 30S subunit.

**Figure 22:**
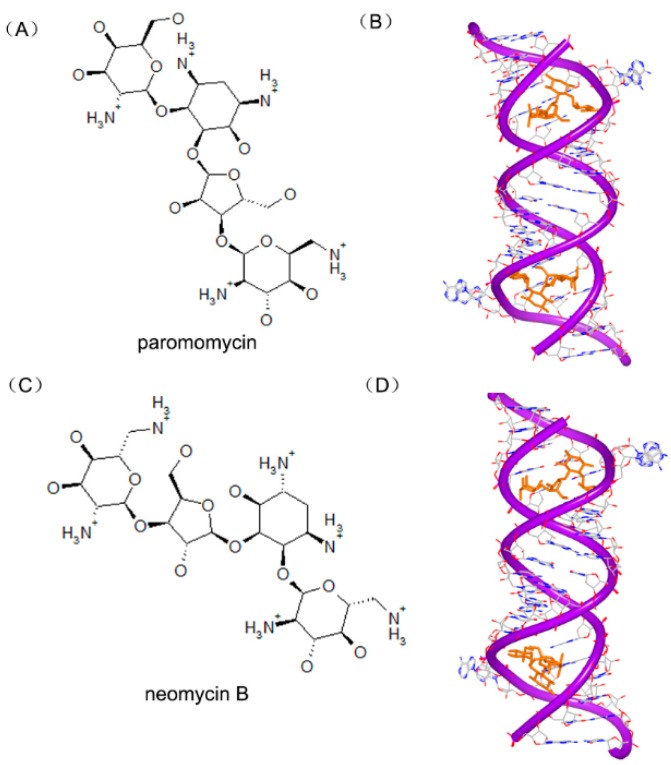
(**A**) Chemical structure of paromomycin; (**B**) complex of a RNA duplex with paromomycin (PDB code: 1J7T); (**C**) chemical structure of neomycin B; and (**D**) complex of a RNA duplex with neomycin B (PDB code: 2ET4). The orange structure represents the small molecule; the purple structure represents the backbone of nucleic acid; the blue and red atoms in the molecule represent nitrogen and oxygen atoms, respectively.

Other aminoglycoside compounds have also been determined and reported. The paromomycin (Figure 22A) is also called monomycin and aminosidine. The detailed complex of two paromomycin molecules and an RNA fragment was solved. The molecule is located in the deep position of the RNA groove forming hydrogen bonds with water, as shown in Figure 22B [88]. Neomycin B (Figure 22C) is an aminoglycoside antibiotic different with paromomycin. Neomycin B contains an ammonium group not hydroxyl group in the structure. The complex structure (Figure 22D) of neomycin B and RNA duplex shows a similar interaction, compared to other aminoglycosides [86].

### 3.2. Small Molecules Targeting Riboswitches

In molecular biology, a riboswitch is a regulatory segment of a messenger RNA (mRNA) molecule, resulting in a change in production of the proteins encoded by the mRNA. Riboswitch consists of an aptamer domain and an expression platform. In recent years, various kinds of riboswitches have been reported [92,93,94,95]. Because riboswitch can bind a ligand naturally, it is an ideal target for anti-bacterial agent.

**Figure 23:**
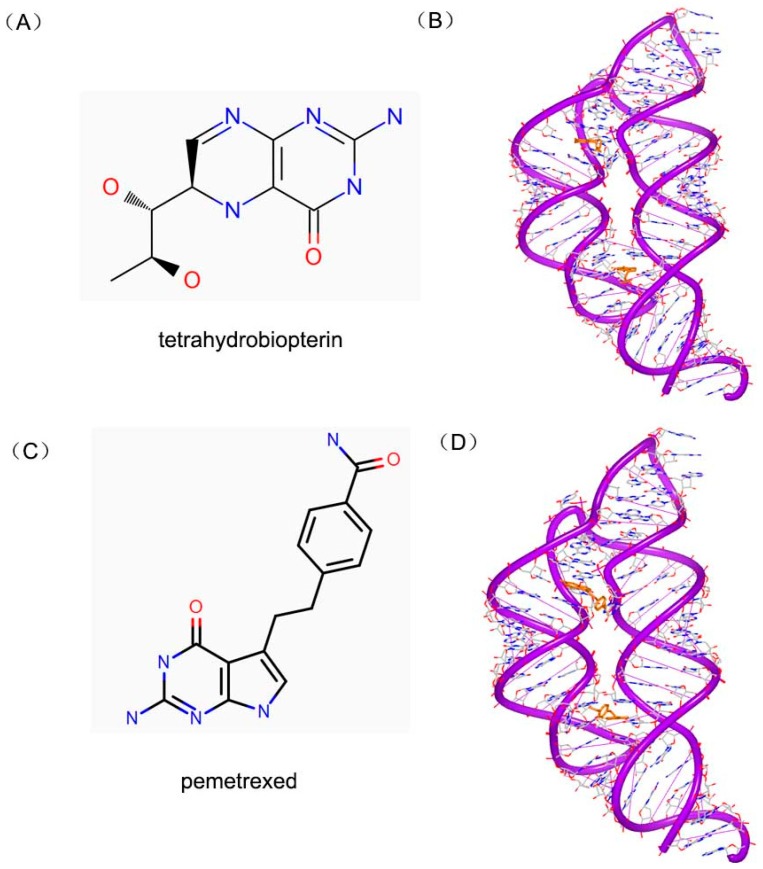
(**A**) Chemical structure of tetrahydrobiopterin; (**B**) complex of a RNA duplex with tetrahydrobiopterin (PDB code: 4LVX); (**C**) chemical structure of pemetrexe; and (**D**) complex of a RNA duplex with pemetrexed (PDB code: 4LVY). The orange structure represents the small molecule; the purple structure represents the backbone of nucleic acid; the blue and red atoms in the molecule represent nitrogen and oxygen atoms, respectively.

The tetrahydrofolate (THF) riboswitch can regulate the transportation and metabolism of folate through combining THF molecules. This riboswitch was identified to bind various kinds of folates, such as dihydrofolate (DHF) and tetrahydrobiopterin (BH_4_). Tetrahydrobiopterin (Figure 23A), for example, is an important cofactor of the hydroxylase enzymes of aromatic amino acid. The complex of BH_4_ and THF riboswitch (Figure 23B) indicates that the placement of the pterin ring in BH4 is identical to that of THF. Pemetrexed (Figure 23C) is a structural analog with folic acid and is also used as chemotherapeutic drug in treatment. The crystal complex of riboswitch and pemetrexed indicates that the compound binds to the RNA structure almost identically as THF, as shown in Figure 23D [96]. Both structures reveal that reduced folates are primarily recognized by the RNA through their pterin moiety.

**Figure 24:**
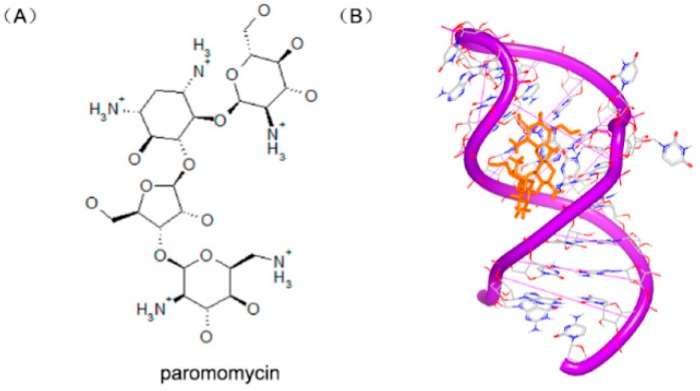

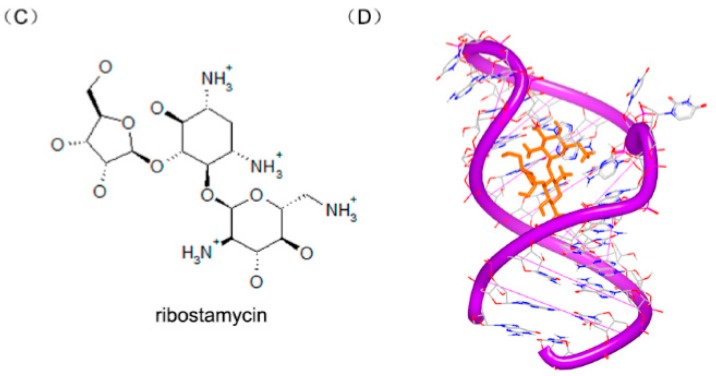
(**A**) Chemical structure of paromomycin; (**B**) complex of a neomycin riboswitch RNA with paromomycin (PDB code: 2MXS); (**C**) chemical structure of ribostamycin; and (**D**) complex of a neomycin riboswitch RNA with ribostamycin (PDB code: 2N0J). The orange structure represents the small molecule; the purple structure represents the backbone of nucleic acid; the blue and red atoms in the molecule represent nitrogen and oxygen atoms, respectively.

Recently, some aminoglycosides are identified to bind with the riboswitch RNA, including ribostamycin and paromomycin [97].To understand the biochemical elements for the significant riboswitch specificity, the complexes of paromomycin (Figure 24A) and ribostamycin (Figure 24C) with neomycin riboswitch RNA were resolved. The complexes of the two structures indicate that the two helix structures of the rRNA formed a consecutive A-form helical structure through stacking between two G–C base pairs, as shown in Figure 24B,D. In the two complex structures, the 6′ substructures of the two molecules locate at a close area, near the hinge region between the upper and the lower helix stem.

### 3.3. Small Molecules Targeting MicroRNA

MicroRNAs (miRNA), a class of noncoding RNA molecules, contain ~22 length nucleotides, which function for gene expression [98,99,100]. In order to understand the structure of miRNA and the interaction between miRNA and small molecules, computational high throughput screenings play an important role, because computers could drastically speed up the identification and optimization of compounds targeting miRNA, compared to traditional drug discovery strategy.

**Figure 25:**
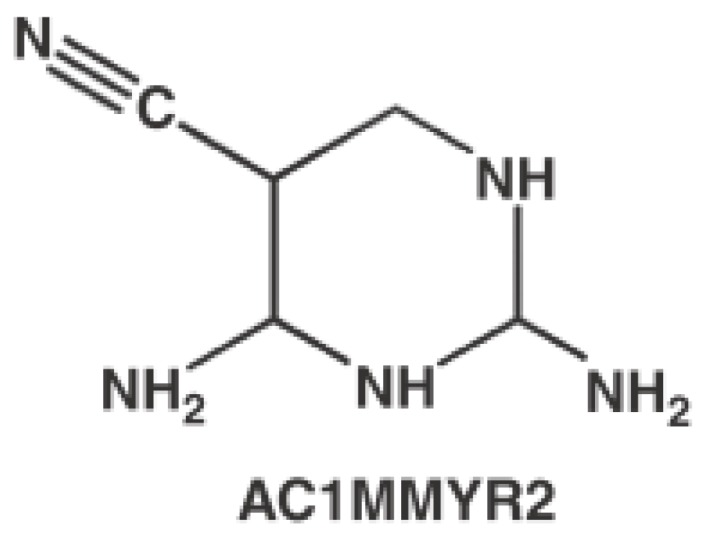
Chemical structure of AC1MMYR2.

Recently, Shi *et al.* discovered that AC1MMYR2 (Figure 25) was an efficient and selective inhibitor of microRNA-21 through *in silicon* virtual screening [101]. The pre-miRNA-21 was modeled by MC-Fold/MC-Sym [102] pipeline. Forty-eight predicted compounds were selected by AutoDock. Finally, AC1MMYR2 was identified to inhibit tumor proliferation and migration.

### 3.4. Small Molecules Targeting Long Non-Coding RNA

**Figure 26:**
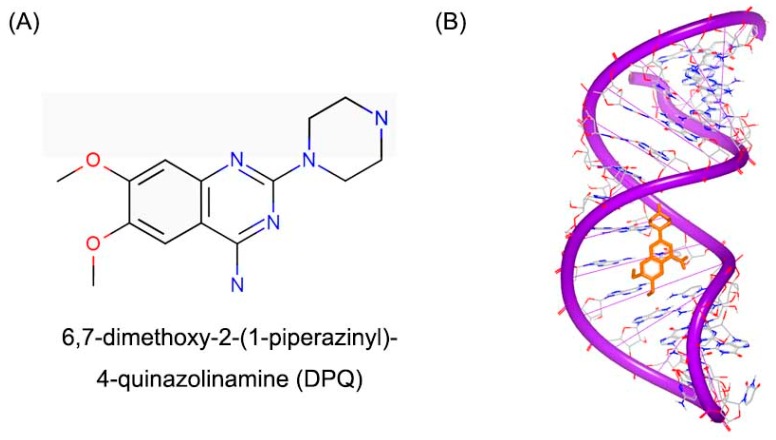
(**A**) Chemical structure of DPQ; and (**B**) structure of a RNA promoter complex with DPQ (PDB code: 2LWK). The orange structure represents the small molecule; the purple structure represents the backbone of nucleic acid; the blue and red atoms in the molecule represent nitrogen and oxygen atoms, respectively.

Long noncoding RNA (lncRNA) has been shown to play important functional roles in development and disease processes [103,104,105,106]. Influenza A virus contains eight separated, single-stranded RNAs that encode 13 kinds of proteins. The RNA dependent RNA polymerase (RdRp) can recognize a specific RNA conformation, lncRNA in particular, regulating the beginning of replication and transcription process [107,108]. A small molecule (DPQ, Figure 26A) was found to bind with the lncRNA [109]. The crystal structure showed small molecule locates in the major groove of the (A–A)–U loop (Figure 26B). The bindings of small molecule widen the major groove close the position of G14–C21 and G13–C22 base pairs.

## 4. Conclusions

Nucleic acid drug discovery is a sophisticated system and comprises of a large number of stages, including target selection, binding site verification, potent inhibitor screening, and compound optimization. Small molecule targeting nucleic acid is still a tremendous challenge. Due to the low specificity of DNA or RNA binders, high rate of the failure in clinical trials is the obstacle of nucleic acid drug discovery. The progress of nucleic acid drug discovery needs the development of structural biology and other related technologies.

DNAs as the fundamental target of various small molecules, for instance antibiotics and antitumor agents, have been verified. It is obvious that small molecules bind with different mechanisms to different DNA conformation. Small molecules can bind to DNA duplex in various kinds of mechanisms, including covalent binding, insertion, and some multifunction. For triplex and quadruplex, however, the intercalation or insertion is the common binding mode for small molecules. In particular, the majority of such small molecules contain planar aromatic ring systems. Such substructure can keep the complex more stable.

RNA attracts less attention than DNA targets. Nevertheless, recently, much progress has been made in discovering small molecule inhibitor stargeting to RNA with high specificity and bioactivity. *In silico* virtual screening for novel inhibitors targeting RNA has produced original small molecule RNA binders.

In summary, small molecules targeting nucleic acids can regulate a large number of biological processes. However, major challenges and issues are yet to be resolved. Therefore, the study and development of promising small molecules targeting nucleic acids strategies is ongoing.

## References

[1] Bayne E.H., Allshire R.C. (2005). RNA-directed transcriptional gene silencing in mammals. Trends Genet..

[2] Fedor M.J., Williamson J.R. (2005). The catalytic diversity of RNAs. Nat. Rev. Mol. Cell Biol..

[3] Johnstone O., Lasko P. (2001). Translational regulation and RNA localization in drosophila oocytes and embryos. Annu. Rev. Genet..

[4] Smith A.M., Fuchs R.T., Grundy F.J., Henkin T.M. (2010). Riboswitch RNAs: Regulation of gene expression by direct monitoring of a physiological signal. RNA Biol..

[5] Wu L., Belasco J.G. (2008). Let me count the ways: Mechanisms of gene regulation by miRNAs and siRNAs. Mol. Cell.

[6] Gurung R.L., Lim H.K., Venkatesan S., Lee P.S., Hande M.P. (2014). Targeting DNA-PKcs and telomerase in brain tumour cells. Mol. Cancer.

[7] Bottini A., De S.K., Wu B., Tang C., Varani G., Pellecchia M. (2015). Targeting influenza a virus RNA promoter. Chem. Biol. Drug Des..

[8] Cruz J.A., Westhof E. (2009). The dynamic landscapes of RNA architecture. Cell.

[9] Fulle S., Gohlke H. (2010). Molecular recognition of RNA: Challenges for modelling interactions and plasticity. J. Mol. Recognit..

[10] Hermann T. (2002). Rational ligand design for RNA: The role of static structure and conformational flexibility in target recognition. Biochimie.

[11] Brown R.F., Andrews C.T., Elcock A.H. (2015). Stacking free energies of all DNA and RNA nucleoside pairs and dinucleoside-monophosphates computed using recently revised amber parameters and compared with experiment. J. Chem. Theory Comput..

[12] Chauvot de Beauchene I., de Vries S.J., Zacharias M. (2016). Binding site identification and flexible docking of single stranded RNA to proteins using a fragment-based approach. PLoS Comput. Biol..

[13] Spielmann H.P., Dwyer T.J., Hearst J.E., Wemmer D.E. (1995). Solution structures of psoralen monoadducted and cross-linked DNA oligomers by NMR spectroscopy and restrained molecular dynamics. Biochemistry.

[14] Mace K., Aguilar F., Wang J.S., Vautravers P., Gomez-Lechon M., Gonzalez F.J., Groopman J., Harris C.C., Pfeifer A.M. (1997). Aflatoxin b1-induced DNA adduct formation and p53 mutations in CYP450-expressing human liver cell lines. Carcinogenesis.

[15] Brown K.L., Voehler M.W., Magee S.M., Harris C.M., Harris T.M., Stone M.P. (2009). Structural perturbations induced by the α-anomer of the aflatoxin b(1) formamidopyrimidine adduct in duplex and single-strand DNA. J. Am. Chem. Soc..

[16] Noll D.M., Noronha A.M., Wilds C.J., Miller P.S. (2004). Preparation of interstrand cross-linked DNA oligonucleotide duplexes. Front. Biosci..

[17] Swenson M.C., Paranawithana S.R., Miller P.S., Kielkopf C.L. (2007). Structure of a DNA repair substrate containing an alkyl interstrand cross-link at 1.65 Å resolution. Biochemistry.

[18] Huang H., Dooley P.A., Harris C.M., Harris T.M., Stone M.P. (2009). Differential base stacking interactions induced by trimethylene interstrand DNA cross-links in the 5′-CpG-3′ and 5′-GpC-3′ sequence contexts. Chem. Res. Toxicol..

[19] Iyer V.N., Szybalski W. (1963). A molecular mechanism of mitomycin action: Linking of complementary DNA strands. Proc. Natl. Acad. Sci. USA.

[20] Sastry M., Fiala R., Lipman R., Tomasz M., Patel D.J. (1995). Solution structure of the monoalkylatedmitomycin c-DNA complex. J. Mol. Biol..

[21] Wartell R.M., Larson J.E., Wells R.D. (1974). Netropsin. A specific probe for A-T regions of duplex deoxyribonucleic acid. J. Biol. Chem..

[22] Zimmer C. (1975). Effects of the antibiotics netropsin and distamycin a on the structure and function of nucleic acids. Prog. Nucleic Acid Res. Mol. Biol..

[23] Arafa R.K., Ismail M.A., Munde M., Wilson W.D., Wenzler T., Brun R., Boykin D.W. (2008). Novel linear triaryl guanidines, *N*-substituted guanidines and potential prodrugs as antiprotozoal agents. Eur. J. Med. Chem..

[24] Nguyen B., Neidle S., Wilson W.D. (2009). A role for water molecules in DNA-ligand minor groove recognition. Acc. Chem. Res..

[25] Wilson W.D., Tanious F.A., Mathis A., Tevis D., Hall J.E., Boykin D.W. (2008). Antiparasitic compounds that target DNA. Biochimie.

[26] Chen X., Mitra S.N., Rao S.T., Sekar K., Sundaralingam M. (1998). A novel end-to-end binding of two netropsins to the DNA decamers d(ccccciiiii)_2_, d(cccbr^5^cciiiii)_2_ and d(cbr^5^cccciiiii)_2_. Nucleic Acids Res..

[27] Kopka M.L., Yoon C., Goodsell D., Pjura P., Dickerson R.E. (1985). The molecular origin of DNA-drug specificity in netropsin and distamycin. Proc. Natl. Acad. Sci. USA.

[28] Nunn C.M., Garman E., Neidle S. (1997). Crystal structure of the DNA decamer d(cgcaattgcg) complexed with the minor groove binding drug netropsin. Biochemistry.

[29] Schultz P.G., Dervan P.B. (1984). Distamycin and penta-*N*-methylpyrrolecarboxamide binding sites on native DNA. A comparison of methidiumpropyl-EDTA-Fe(II) footprinting and DNA affinity cleaving. J. Biomol. Struct. Dyn..

[30] Tevis D.S., Kumar A., Stephens C.E., Boykin D.W., Wilson W.D. (2009). Large, sequence-dependent effects on DNA conformation by minor groove binding compounds. Nucleic Acids Res..

[31] Chenoweth D.M., Dervan P.B. (2009). Allosteric modulation of DNA by small molecules. Proc. Natl. Acad. Sci. USA.

[32] Moreno T., Pous J., Subirana J.A., Campos J.L. (2010). Coiled-coil conformation of a pentamidine-DNA complex. Acta Crystallogr. D Biol. Crystallogr..

[33] Willis B., Arya D.P. (2006). An expanding view of aminoglycoside-nucleic acid recognition. Adv. Carbohydr. Chem. Biochem..

[34] Goldberg I.H. (1991). Mechanism of neocarzinostatin action: Role of DNA microstructure in determination of chemistry of bistranded oxidative damage. Acc. Chem. Res..

[35] Kappen L.S., Goldberg I.H. (1992). Neocarzinostatin acts as a sensitive probe of DNA microheterogeneity: Switching of chemistry from C-1′ to C-4′ by a C.T mismatch 5′ to the site of DNA damage. Proc. Natl. Acad. Sci. USA.

[36] Myers A.G., Proteau P.J., Handel T.M. (1988). Stereochemical assignment of neocarzinostatin chromophore. Structures of neocarzinostatin chromophore-methyl thioglycolate adducts. J. Am. Chem. Soc..

[37] Gao X., Stassinopoulos A., Ji J., Kwon Y., Bare S., Goldberg I.H. (2002). Induced formation of a DNA bulge structure by a molecular wedge ligand-postactivated neocarzinostatin chromophore. Biochemistry.

[38] Kwon Y., Xi Z., Kappen L.S., Goldberg I.H., Gao X. (2003). New complex of post-activated neocarzinostatin chromophore with DNA: Bulge DNA binding from the minor groove. Biochemistry.

[39] Chaires J.B. (1997). Energetics of drug-DNA interactions. Biopolymers.

[40] Lerman L.S. (1961). Structural considerations in the interaction of DNA and acridines. J. Mol. Biol..

[41] Richards A.D., Rodger A. (2007). Synthetic metallomolecules as agents for the control of DNA structure. Chem. Soc. Rev..

[42] Snyder R.D. (2007). Assessment of atypical DNA intercalating agents in biological and in silico systems. Mutat. Res..

[43] Snyder R.D., McNulty J., Zairov G., Ewing D.E., Hendry L.B. (2005). The influence of *N*-dialkyl and other cationic substituents on DNA intercalation and genotoxicity. Mutat. Res..

[44] Tse W.C., Boger D.L. (2004). Sequence-selective DNA recognition: Natural products and nature’s lessons. Chem. Biol..

[45] Weiss R.B. (1992). The anthracyclines: Will we ever find a better doxorubicin?. Semin. Oncol..

[46] Davies D.B., Eaton R.J., Baranovsky S.F., Veselkov A.N. (2000). NMR investigation of the complexation of daunomycin with deoxytetranucleotides of different base sequence in aqueous solution. J. Biomol. Struct. Dyn..

[47] Frederick C.A., Williams L.D., Ughetto G., van der Marel G.A., van Boom J.H., Rich A., Wang A.H. (1990). Structural comparison of anticancer drug-DNA complexes: Adriamycin and daunomycin. Biochemistry.

[48] Leonard G.A., Brown T., Hunter W.N. (1992). Anthracycline binding to DNA. High-resolution structure of d(tgtaca) complexed with 4′-epiadriamycin. Eur. J. Biochem..

[49] Moore M.H., Hunter W.N., d’Estaintot B.L., Kennard O. (1989). DNA-drug interactions. The crystal structure of d(CGATCG) complexed with daunomycin. J. Mol. Biol..

[50] Wang A.H., Gao Y.G., Liaw Y.C., Li Y.K. (1991). Formaldehyde cross-links daunorubicin and DNA efficiently: HPLC and X-ray diffraction studies. Biochemistry.

[51] Wang A.H., Ughetto G., Quigley G.J., Rich A. (1987). Interactions between an anthracycline antibiotic and DNA: Molecular structure of daunomycin complexed to d(CpGpTpApCpG) at 1.2-A resolution. Biochemistry.

[52] Lambert B., Jones B.K., Roques B.P., Le Pecq J.B., Yeung A.T. (1989). The noncovalent complex between DNA and the bifunctional intercalator ditercalinium is a substrate for the UvrABC endonuclease of *Escherichia coli*. Proc. Natl. Acad. Sci. USA.

[53] Lambert B., Roques B.P., Le Pecq J.B. (1988). Induction of an abortive and futile DNA repair process in *E. coli* by the antitumor DNA bifunctionalintercalator, ditercalinium: Role in *polA* in death induction. Nucleic Acids Res..

[54] Lambert B., Segal-Bendirdjian E., Esnault C., Le Pecq J.B., Roques B.P., Jones B., Yeung A.T. (1990). Recognition by the DNA repair system of DNA structural alterations induced by reversible drug-DNA interactions. Anticancer Drug Des..

[55] Garbay-Jaureguiberry C., Laugaa P., Delepierre M., Laalami S., Muzard G., Le Pecq J.B., Roques B.P. (1987). DNA bis-intercalators as new anti-tumour agents: Modulation of the anti-tumour activity by the linking chain rigidity in the ditercalinium series. Anticancer Drug Des..

[56] Leon P., Garbay-Jaureguiberry C., Barsi M.C., Le Pecq J.B., Roques B.P. (1987). Modulation of the antitumor activity by methyl substitutions in the series of 7H-pyridocarbazole monomers and dimers. J. Med. Chem..

[57] Pelaprat D., Delbarre A., Le Guen I., Roques B.P., Le Pecq J.B. (1980). DNA intercalating compounds as potential antitumor agents. 2. Preparation and properties of 7H-pyridocarbazole dimers. J. Med. Chem..

[58] Gao Q., Williams L.D., Egli M., Rabinovich D., Chen S.L., Quigley G.J., Rich A. (1991). Drug-induced DNA repair: X-ray structure of a DNA-ditercalinium complex. Proc. Natl. Acad. Sci. USA.

[59] Bonjean K., De Pauw-Gillet M.C., Defresne M.P., Colson P., Houssier C., Dassonneville L., Bailly C., Greimers R., Wright C., Quetin-Leclercq J. (1998). The DNA intercalating alkaloid cryptolepine interferes with topoisomerase II and inhibits primarily DNA synthesis in B16 melanoma cells. Biochemistry.

[60] Lisgarten J.N., Coll M., Portugal J., Wright C.W., Aymami J. (2002). The antimalarial and cytotoxic drug cryptolepine intercalates into DNA at cytosine-cytosine sites. Nat. Struct. Biol..

[61] Bailly C., Henichart J.P. (1991). DNA recognition by intercalator-minor-groove binder hybrid molecules. Bioconjug. Chem..

[62] Banerjee D., Pal S.K. (2007). Simultaneous binding of minor groove binder and intercalator to dodecamer DNA: Importance of relative orientation of donor and acceptor in FRET. J. Phys. Chem. B.

[63] Rettig M., Weingarth M., Langel W., Kamal A., Kumar P.P., Weisz K. (2009). Solution structure of a covalently bound pyrrolo[2,1-*c*][1,4]benzodiazepine-benzimidazole hybrid to a 10mer DNA duplex. Biochemistry.

[64] Rettig M., Langel W., Kamal A., Weisz K. (2010). NMR structural studies on the covalent DNA binding of a pyrrolobenzodiazepine-naphthalimide conjugate. Org. Biomol. Chem..

[65] Le Doan T., Perrouault L., Praseuth D., Habhoub N., Decout J.L., Thuong N.T., Lhomme J., Helene C. (1987). Sequence-specific recognition, photocrosslinking and cleavage of the DNA double helix by an oligo-[α]-thymidylate covalently linked to an azidoproflavine derivative. Nucleic Acids Res..

[66] Moser H.E., Dervan P.B. (1987). Sequence-specific cleavage of double helical DNA by triple helix formation. Science.

[67] Jain A., Wang G., Vasquez K.M. (2008). DNA triple helices: Biological consequences and therapeutic potential. Biochimie.

[68] Sandstrom K., Warmlander S., Bergman J., Engqvist R., Leijon M., Graslund A. (2004). The influence of intercalator binding on DNA triplex stability: Correlation with effects on A-tract duplex structure. J. Mol. Recognit..

[69] Arya D.P., Micovic L., Charles I., Coffee R.L., Willis B., Xue L. (2003). Neomycin binding to Watson–hoogsteen (W–H) DNA triplex groove: A model. J. Am. Chem. Soc..

[70] Xue L., Charles I., Arya D.P. (2002). Pyrene-neomycin conjugate: Dual recognition of a DNA triple helix. Chem. Commun. (Camb.).

[71] Dapic V., Bates P.J., Trent J.O., Rodger A., Thomas S.D., Miller D.M. (2002). Antiproliferative activity of G-quartet-forming oligonucleotides with backbone and sugar modifications. Biochemistry.

[72] Harrison R.J., Gowan S.M., Kelland L.R., Neidle S. (1999). Human telomerase inhibition by substituted acridine derivatives. Bioorg. Med. Chem. Lett..

[73] Heald R.A., Modi C., Cookson J.C., Hutchinson I., Laughton C.A., Gowan S.M., Kelland L.R., Stevens M.F. (2002). Antitumor polycyclic acridines. 8.(1) Synthesis and telomerase-inhibitory activity of methylated pentacyclic acridinium salts. J. Med. Chem..

[74] Riou J.F., Guittat L., Mailliet P., Laoui A., Renou E., Petitgenet O., Megnin-Chanet F., Helene C., Mergny J.L. (2002). Cell senescence and telomere shortening induced by a new series of specific G-quadruplex DNA ligands. Proc. Natl. Acad. Sci. USA.

[75] Sun D., Thompson B., Cathers B.E., Salazar M., Kerwin S.M., Trent J.O., Jenkins T.C., Neidle S., Hurley L.H. (1997). Inhibition of human telomerase by a G-quadruplex-interactive compound. J. Med. Chem..

[76] Clark G.R., Pytel P.D., Squire C.J., Neidle S. (2003). Structure of the first parallel DNA quadruplex-drug complex. J. Am. Chem. Soc..

[77] Martino L., Virno A., Pagano B., Virgilio A., Di Micco S., Galeone A., Giancola C., Bifulco G., Mayol L., Randazzo A. (2007). Structural and thermodynamic studies of the interaction of distamycin a with the parallel quadruplex structure [d(tggggt)]_4_. J. Am. Chem. Soc..

[78] Neidle S. (2009). The structures of quadruplex nucleic acids and their drug complexes. Curr. Opin. Struct. Biol..

[79] Comartin D.J., Brown E.D. (2006). Non-ribosomal factors in ribosome subunit assembly are emerging targets for new antibacterial drugs. Curr. Opin. Pharmacol..

[80] Connolly K., Culver G. (2009). Deconstructing ribosome construction. Trends Biochem. Sci..

[81] Franceschi F., Duffy E.M. (2006). Structure-based drug design meets the ribosome. Biochem. Pharmacol..

[82] Poehlsgaard J., Douthwaite S. (2005). The bacterial ribosome as a target for antibiotics. Nat. Rev. Microbiol..

[83] Yonath A. (2005). Antibiotics targeting ribosomes: Resistance, selectivity, synergism and cellular regulation. Annu. Rev. Biochem..

[84] Aboul-ela F. (2010). Strategies for the design of RNA-binding small molecules. Future Med. Chem..

[85] Thomas J.R., Hergenrother P.J. (2008). Targeting RNA with small molecules. Chem. Rev..

[86] Francois B., Russell R.J., Murray J.B., Aboul-ela F., Masquida B., Vicens Q., Westhof E. (2005). Crystal structures of complexes between aminoglycosides and decoding a site oligonucleotides: Role of the number of rings and positive charges in the specific binding leading to miscoding. Nucleic Acids Res..

[87] Francois B., Szychowski J., Adhikari S.S., Pachamuthu K., Swayze E.E., Griffey R.H., Migawa M.T., Westhof E., Hanessian S. (2004). Antibacterial aminoglycosides with a modified mode of binding to the ribosomal-RNA decoding site. Angew. Chem. Int. Ed. Engl..

[88] Vicens Q., Westhof E. (2001). Crystal structure of paromomycin docked into the eubacterial ribosomal decoding a site. Structure.

[89] Vicens Q., Westhof E. (2002). Crystal structure of a complex between the aminoglycoside tobramycin and an oligonucleotide containing the ribosomal decoding a site. Chem. Biol..

[90] Vicens Q., Westhof E. (2003). Crystal structure of geneticin bound to a bacterial 16s ribosomal RNA a site oligonucleotide. J. Mol. Biol..

[91] Kondo J., Francois B., Russell R.J., Murray J.B., Westhof E. (2006). Crystal structure of the bacterial ribosomal decoding site complexed with amikacin containing the γ-amino-α-hydroxybutyryl (haba) group. Biochimie.

[92] Blount K.F., Breaker R.R. (2006). Riboswitches as antibacterial drug targets. Nat. Biotechnol..

[93] Deigan K.E., Ferre-D’Amare A.R. (2011). Riboswitches: Discovery of drugs that target bacterial gene-regulatory RNAs. Acc. Chem. Res..

[94] Mulhbacher J., St-Pierre P., Lafontaine D.A. (2010). Therapeutic applications of ribozymes and riboswitches. Curr. Opin. Pharmacol..

[95] Zhang J., Lau M.W., Ferre-D’Amare A.R. (2010). Ribozymes and riboswitches: Modulation of RNA function by small molecules. Biochemistry.

[96] Trausch J.J., Batey R.T. (2014). A disconnect between high-affinity binding and efficient regulation by antifolates and purines in the tetrahydrofolate riboswitch. Chem. Biol..

[97] Duchardt-Ferner E., Gottstein-Schmidtke S.R., Weigand J.E., Ohlenschlager O., Wurm J.P., Hammann C., Suess B., Wohnert J. (2016). What a difference an OH makes: Conformational dynamics as the basis for the ligand specificity of the neomycin-sensing riboswitch. Angew. Chem. Int. Ed..

[98] Ambros V. (2003). MicroRNA pathways in flies and worms: Growth, death, fat, stress, and timing. Cell.

[99] Bartel D.P. (2004). MicroRNAs: Genomics, biogenesis, mechanism, and function. Cell.

[100] Lai E.C. (2003). MicroRNAs: Runts of the genome assert themselves. Curr. Biol..

[101] Shi Z., Zhang J., Qian X., Han L., Zhang K., Chen L., Liu J., Ren Y., Yang M., Zhang A. (2013). AC1MMYR2, an inhibitor of dicer-mediated biogenesis of oncomir miR-21, reverses epithelial-mesenchymal transition and suppresses tumor growth and progression. Cancer Res..

[102] Parisien M., Major F. (2008). The MC-Fold and MC-Sym pipeline infers RNA structure from sequence data. Nature.

[103] Brown C.J., Ballabio A., Rupert J.L., Lafreniere R.G., Grompe M., Tonlorenzi R., Willard H.F. (1991). A gene from the region of the human X inactivation centre is expressed exclusively from the inactive X chromosome. Nature.

[104] Gupta R.A., Shah N., Wang K.C., Kim J., Horlings H.M., Wong D.J., Tsai M.C., Hung T., Argani P., Rinn J.L. (2010). Long non-coding RNA hotair reprograms chromatin state to promote cancer metastasis. Nature.

[105] Lee M.P., DeBaun M.R., Mitsuya K., Galonek H.L., Brandenburg S., Oshimura M., Feinberg A.P. (1999). Loss of imprinting of a paternally expressed transcript, with antisense orientation to K_V_LQT1, occurs frequently in beckwith–wiedemann syndrome and is independent of insulin-like growth factor II imprinting. Proc. Natl. Acad. Sci. USA.

[106] Pasmant E., Laurendeau I., Heron D., Vidaud M., Vidaud D., Bieche I. (2007). Characterization of a germ-line deletion, including the entire *INK4/ARF* locus, in a melanoma-neural system tumor family: Identification of *ANRIL*, an antisense noncoding RNA whose expression coclusters with *ARF*. Cancer Res..

[107] Brownlee G.G., Sharps J.L. (2002). The RNA polymerase of influenza a virus is stabilized by interaction with its viral RNA promoter. J. Virol..

[108] Desselberger U., Racaniello V.R., Zazra J.J., Palese P. (1980). The 3′ and 5′-terminal sequences of influenza A, B and C virus RNA segments are highly conserved and show partial inverted complementarity. Gene.

[109] Lee M.K., Bottini A., Kim M., Bardaro M.F., Zhang Z., Pellecchia M., Choi B.S., Varani G. (2014). A novel small-molecule binds to the influenza a virus RNA promoter and inhibits viral replication. Chem. Commun. (Camb.).

